# Traditional Chinese Medicine Extract from Huaier Increases the Expression of Duffy Antigen Receptor for Chemokines and Reduces the Expression of Its Ligands

**DOI:** 10.1155/2018/6756092

**Published:** 2018-07-24

**Authors:** Ying Chen, Qianjun Chen, Fengfeng Xie, Hui Peng, Yanlan Wu, Shaowen Zhong, Zhiyu Wang, Wenxia Li, Wanjun Xie

**Affiliations:** ^1^Department of Breast Surgery, Guangdong Traditional Chinese Medicine Hospital, Guangzhou, Guangdong 510000, China; ^2^Department of Pathology, Guangdong Traditional Chinese Medicine Hospital, Guangzhou, Guangdong 510000, China; ^3^Department of Anesthesia, Guangdong Traditional Chinese Medicine Hospital, Guangzhou, Guangdong 510000, China

## Abstract

**Aims:**

The aim of the present study is to investigate whether the aqueous extract from Huaier, a traditional Chinese medicine (TCM), can affect the expression of Duffy antigen receptor for chemokines (DARC) and its ligands. Moreover, we compare the status of DARC in primary and metastatic breast cancer tissues from the same patient.

**Methods:**

Immunohistochemistry was used to detect the expression of DARC in primary and metastatic focuses in 30 patients with breast cancer. The effect of Huaier aqueous extract on the expression of DARC and its ligands was investigated by quantitative real-time polymerase chain reaction, Western blotting, and enzyme-linked immunosorbent assay.

**Results:**

The expression score of DARC in primary focuses was significantly higher than that in metastatic focuses, while changes of ER, PR, and HER2 receptors were not significantly different between primary and metastatic focuses. Huaier aqueous extract promoted the expression of DARC and reduced the secretion of CC chemokine ligand 2 (CCL-2), CXC chemokine ligand 8 (CXCL-8, IL-8), matrix metalloproteinase 2 (MMP-2), and CXC chemokine ligand 1 (CXCL-1).

**Conclusion:**

The present study demonstrates that difference in expression level of DARC between primary and metastatic focuses of breast cancer was significant, while differences in expression of ER, PR, and HER2 between primary and metastatic focuses were not significant. DARC may play a negative role in the metastasis of breast cancer. Traditional Chinese medicine extract from Huaier can increase DARC expression and reduce the expression of its ligands such as CCL-2, IL-8, MMP-2, and CXCL-1.

## 1. Introduction

Breast cancer is the most severe malignant tumor in women in China [1]. The major cause of mortality from breast cancer is metastasis to distant organs. However, the 5-year survival rate of patients with metastatic breast cancer is still around 25%, and the median survival period is about 2–3 years [2, 3].

Chemokines are a class of specific small-molecule proteins that play important roles in the recruitment and activation of leukocytes. They have also been highlighted in cancer progression and metastasis, as well as the regulation of host immune responses. There are some special chemokine receptors that are incapable of transmitting their signals through classic G protein-mediated pathways. Therefore, these receptors act as scavengers by efficiently internalizing their cognate chemokine ligands [4].

Duffy antigen receptor for chemokines (DARC) is a typical chemokine decoy receptor that is first discovered in hemophiliacs. It is widely expressed on erythrocytes and vascular endothelial cells. DARC can bind to chemokines that are associated with angiogenesis. Since DARC lacks 7 transmembrane glycoproteins, DARC does not cause cell signal transduction or cell metabolism after binding with its ligands [5]. It has been shown that DARC is involved as a negative regulator in the pathogenesis of common cancers, mainly by sequestrating promalignant chemokines. Several studies show that downregulation of DARC indicates poor prognosis of breast cancer, colorectal cancer, prostate cancer, cervical squamous cell cancer, thyroid cancer, and gastric cancer [6–10].

Hormone receptor-positive MCF-7 cells express high levels of DARC, and triple negative MDA-MB-231 cells express low levels of DARC. The biological behaviors of hormone receptor-positive cancers seem to be more gentle than those of triple negative cancers, and the expression level of DARC is associated with the grade of malignancy. When enhancing DARC expression by gene transfection, cell growth and metastasis potential are reduced [10]. If there is one way to enhance DARC expression by an extraneous factor, it can be an important method to reduce breast cancer metastasis.

Traditional Chinese medicine, a rich source of potent anticancer agents, is attracting increasing attention worldwide. Recently, the anticancer activity of *Trametes robiniophila* Murr. (Huaier) has been widely investigated. Huaier is isolated from the extract of officinal fungi that has been used in traditional Chinese medicine for approximately 1600 years, and proteoglycan has been identified as the effective ingredient [11]. It has been widely used in the treatment of breast cancer, liver cancer, prostate cancer, and gynecological malignant tumors [12–14].

Previous studies show that Huaier aqueous extract can inhibit the proliferation of breast cancer cells by inducing apoptosis, suppress tumor metastasis, induce autophagic cell deaths, disturb DNA repair, and stimulate the ability of T and B lymphocytes [11, 15, 16]. However, the mechanisms are not fully understood yet. Whether Huaier aqueous extract affects the expression of chemokines and DARC has not been investigated. To test our hypothesis, we compare the expression of DARC in primary and metastatic breast cancer. Then, we investigate the effect of Huaier aqueous extract on the expression of DARC and its ligand chemokines.

## 2. Materials and Methods

### 2.1. Patients

A total of 30 female patients with breast cancer were selected from Guangdong Traditional Chinese Medicine Hospital (Guangzhou, China) for the study. Tumor specimens were all invasive ductal carcinomas, according to the WHO tumor classification. The mean age of patients with primary breast cancer was 50.33 years (ranging from 29 to 69 years). However, average age of these patients when recurrence and metastasis occurred was 52.9 years (ranging from 34 to 71 years). The mean disease-free survival (DFS) time was 2.6 years (ranging from 1 to 9 years). According to the clinicopathological surrogate definitions of subtypes which were adopted by the 13th St. Gallen International Breast Cancer Conference (2013) Expert Panel [17], we divided the patients into 4 groups. In addition, 3 cases were Luminal A-like, 11 cases were Luminal B-like (human epidermal growth factor receptor 2- (HER2-) negative), 5 cases were Luminal B-like (HER2-positive), 3 cases were HER2-positive (nonluminal), and 8 cases were triple negative (ductal). Informed consent was obtained from each patient prior to enrollment in the study. All specimens were obtained following informed consent, and procedures were conducted in accordance with Ethical Standards of the Declaration of Helsinki. Written permission was provided by the hospital and local ethics committee of Guangdong Traditional Chinese Medicine Hospital for the study. The primary tumor characteristics are presented in Table 1.

### 2.2. Immunohistochemical Staining

Sections were incubated with goat anti-human anti-DARC polyclonal antibody (1 : 100 dilution; Santa Cruz, CA, USA) at 4°C overnight. ABC peroxidase staining was then employed, according to the manufacturer's instructions (Vector Laboratories, Burlingame, CA, USA). Colorimetric detection was performed with 3,3′-diaminobenzidine (DAB). Positive reactions were defined as those showing brown signals in cell cytoplasm and cytomembrane. For DARC, a staining index (values 0–12) was determined by multiplying the score for staining intensity with the score for positive area. The intensity was scored as follows: 0, negative; 1, weak; 2, moderate; and 3, strong. The frequency of positive cells was defined as follows: 0, less than 5%; 1, 5%–25%; 2, 26%–50%; 3, 51%–75%; and 4, greater than 75%. The last score was intensity score multiplied by frequency score. For ER, PR, and HER2, the determinations were performed by Pathology Department of Guangdong Traditional Chinese Medicine Hospital.

### 2.3. Cells

MDA-MB-231 and MCF-7 cell lines were obtained from American Type Culture Collection (ATCC) (Manassas, VA, USA). MDA-MB-231 cells were maintained in L15 medium supplemented with 10% fetal bovine serum at 37°C in sterile culture dishes. MCF-7 cells were cultured in RPMI-1640 medium supplemented with 10% fetal bovine serum and penicillin-streptomycin (Flow Laboratories, Rockville, MD) at 37°C. All cell lines were certified to be mycoplasma-free. Then, we treated the cells with 4 mg/ml or 8 mg/ml Huaier aqueous extract for 24 h or 48 h.

### 2.4. Quantitative Real-Time Polymerase Chain Reaction (qRT-PCR)

Total RNA was isolated with TRIzol reagent following the manufacturer's manual (Thermo Fisher Scientific, Waltham, MA, USA). RNA was treated with DNase. Total RNA (1 *μ*g) was converted to cDNA using RevertAid First Strand cDNA Synthesis Kit (Thermo Fisher Scientific, Waltham, MA, USA). Specific primers of DARC and other relevant molecules used in the experiments are shown in Table 2. The amplified products were electrophoresed on a 1.2% agarose gel and stained with ethidium bromide.

qRT-PCR was performed using fluorescence temperature cycler (Opticon, MJ Research, St. Bruno, Canada) and SYBR Green PCR core reagent kit according to the manufacturer's instructions (Takara, Dalian, China). An initial incubation of 50°C for 2 min was followed by denaturing at 95°C for 10 sec and then 40 cycles at 95°C for 15 s and 60°C for 1 min. PCR products were detected by bound SYBR Green double-stranded DNA fluorescence, and the comparative threshold cycle (2^−ΔΔcT^) method was used to enable quantification of mRNA of these genes. All samples were tested in triplicate. Target gene expression was compared to the housekeeping gene GAPDH. After PCR, a melting curve was obtained and analyzed.

### 2.5. Enzyme-Linked Immunosorbent Assay (ELISA)

Cells were seeded in six-well plates before being treated with Huaier aqueous extract. After 24 h of growth, 1.5 ml of medium was collected from each well to evaluate the levels of CC chemokine ligand 2 (CCL-2), CXC chemokine ligand 8 (CXCL-8, IL-8), matrix metalloproteinase 2 (MMP-2), and CXC chemokine ligand 1 (CXCL-1) by ELISA. The supernatants from each time point were collected and analyzed for the protein expression using a commercially available ELISA kit (DuoSet, R&D Systems Inc., Minneapolis, MN, USA) according to the manufacturer's instructions. The plates were read at 450 nm. Protein concentrations in conditioned media were calculated from a standard curve generated by adding recombinant to specific unconditioned media.

### 2.6. Western Blotting

Proteins were extracted from cultured cells and then quantitated with the bicinchoninic acid (BCA) assay kit (Pierce, Rockford, IL, USA), using bovine serum albumin as standard. Equal amounts of protein (50 *μ*g) from different cells were separated by 10% SDS-PAGE and then incubated with antibodies against target proteins. Target proteins were detected by enhanced chemiluminescence (ECL) kit (Amersham Pharmacia Biotech, Uppsala, Sweden) and exposed to BioMax ML film (Eastman Kodak, Rochester, NY, USA). Images were captured by Alpha Image 950 documentation system (Alpha Innotech, San Leandro, CA, USA).

### 2.7. Statistical Analysis

Data were expressed as means ± SD. SPSS software 13.0 version (IBM, Armonk, NY, USA) was used for statistical analysis. Difference in the expression of DARC between primary and metastatic tumors was assessed using Pearson's *χ*^2^ test.

## 3. Results

### 3.1. Expression Level of DARC in Primary Breast Cancer Tissues Is Significantly Higher than That in Metastatic Tissues

**Figure 1:**
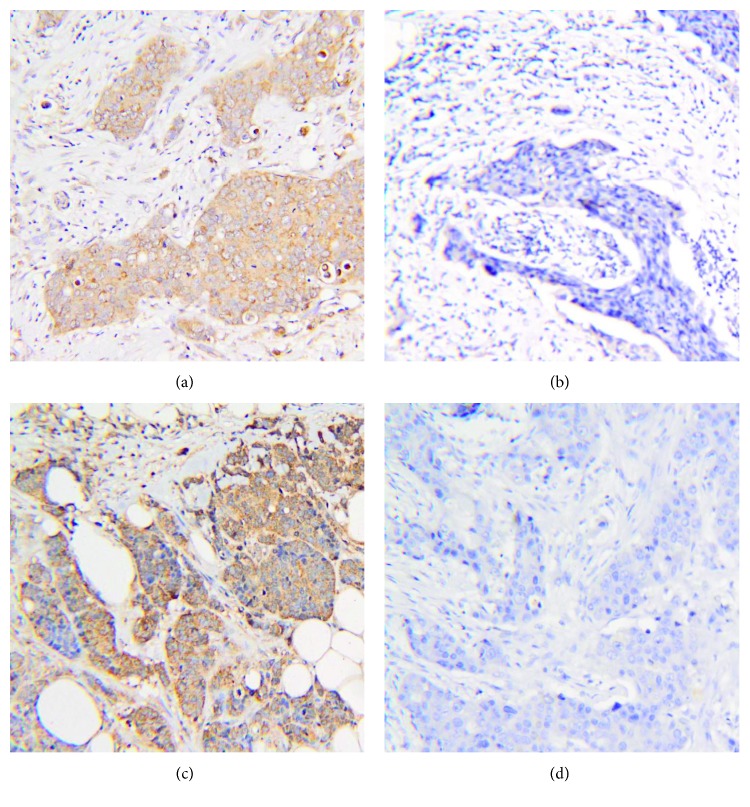
Expression of DARC in human primary and metastatic breast cancer tissue samples with immunohistochemical staining. (a, b) Patient 1: (a) primary tissues (×20, positive 8 scores) and (b) metastatic tissues (×20, negative 0 scores). (c, d) Patient 2: (c) primary tissues (×20, positive 12 scores) and (d) metastatic tissues (×20, negative 0 scores).

Before investigating the expression levels of DARC in primary and metastatic breast cancer tissues, the expressions of HER2, estrogen receptor (ER), and progesterone (PR) were first measured. The expression of HER2 in 3 patients turned from negative in primary tumor tissues to positive in metastatic tumor tissues. ER expression in 9 patients and PR expression in 7 patients were positive in both primary tumor tissues and metastatic tumor tissues. Total discordance rates of HER2, ER, and PR were 30%, 23%, and 10%, respectively, but the expression changes of HER2, ER, and PR were not significantly different between primary and metastatic foci. The expression score of DARC in primary foci was significantly higher than that in metastatic foci (*P* < 0.05) (Figure 1 and Table 3). The result suggests that the expression score of DARC in primary breast cancer tissues is significantly higher than that in metastatic tissues.

### 3.2. Treatment with Huaier Aqueous Extract Enhances the Expression of DARC in MCF-7 and MDA-MB-231 Cells

**Figure 2:**
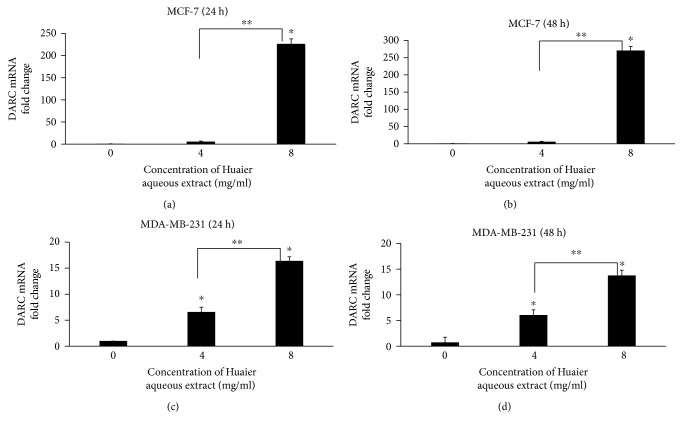
Effect of Huaier aqueous extract on DARC mRNA expression in (a, b) MCF-7 cells and (c, d) MDA-MB-231 cells after treatment with different concentrations (4 or 8 mg/ml) of Huaier aqueous extract for 24 or 48 h. Quantitative real-time polymerase chain reaction was used to measure mRNA expression. ^∗^*P* < 0.05 compared with values at 0 mg/ml. ^∗∗^*P* < 0.05 between 8 mg/ml and 4 mg/ml groups.

**Figure 3:**
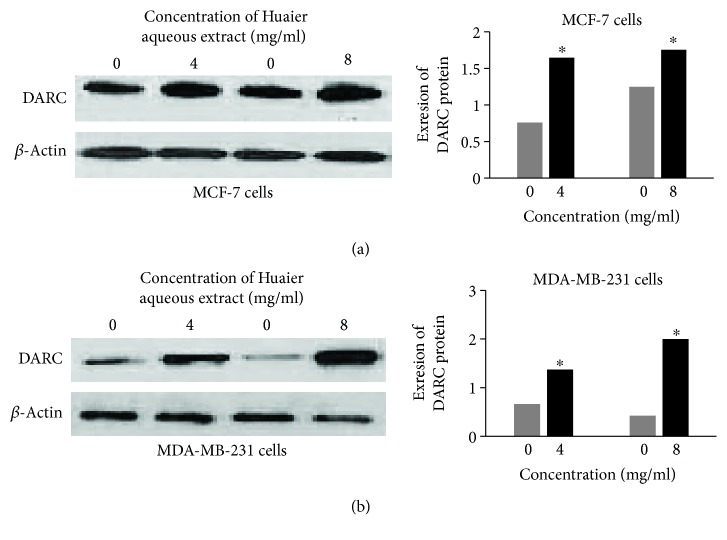
Effect of Huaier aqueous extract on DARC protein expression in (a) MCF-7 cells and (b) MDA-MB-231 cells after treatment with different concentrations (4 or 8 mg/ml) of Huaier aqueous extract for 48 h. Western blotting was used to measure protein expression. The histogram shows the grey level change in different conditions. ^∗^*P* < 0.05 compared with 4 mg/ml group at the same time point.

To investigate the expression changes of DARC after treatment with Huaier aqueous extract, qRT-PCR and Western blotting were performed on MD-MB-231 and MCF-7 cells. The data showed that mRNA expression levels of DARC after treatment with Huaier aqueous extract (4 or 8 mg/ml) for 24 or 48 h were significantly higher than that in control (*P* < 0.05; Figure 2), and protein expression levels of DARC after treatment with Huaier aqueous extract (4 or 8 mg/ml) for 48 h were significantly higher than those in control (*P* < 0.05; Figure 3). The results indicate that treatment with Huaier aqueous extract enhances the expression of DARC in MCF-7 and MDA-MB-231 cells.

### 3.3. The mRNA Expression Levels of the Ligands of DARC Are Reduced by Huaier Aqueous Extract, Especially in MCF-7 Cells

**Figure 4:**
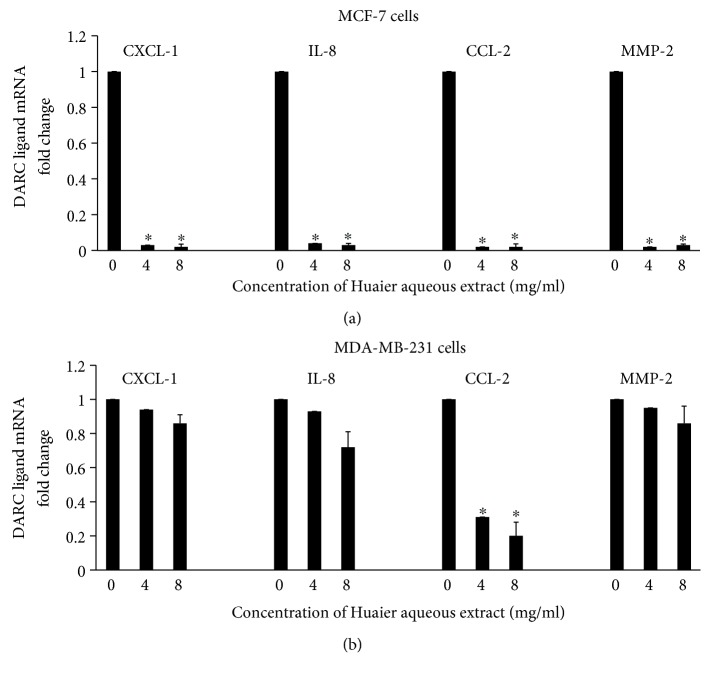
Effect of Huaier aqueous extract on the expression of DARC ligands. Expression of mRNA of CXCL-1, IL-8, CCL-2, and MMP-2 in (a) MCF-7 cells and (b) MDA-MB-231 cells treated by different concentrations (4 or 8 mg/ml) of Huaier aqueous extract for 24 h. Expression of mRNA was assessed by quantitative real-time polymerase chain reaction. ^∗^*P* < 0.05 compared with values at 0 mg/ml.

To study the effect of Huaier aqueous extract on the ligands of DARC in vitro, qRT-PCR was also employed. The data showed that mRNA expression levels of CXCL-1, IL-8, CCL-2, and MMP-2 in MCF-7 cells after treatment with Huaier aqueous extract (4 or 8 mg/ml) for 24 h were significantly lower than that in control (*P* < 0.05) (Figure 4(a)). However, mRNA levels of CCL-2 but not CXCL-1, IL-8, and MMP-2 were significantly reduced by treatment with Huaier aqueous extract (4 or 8 mg/ml) in MDA-MB-231 cells (*P* < 0.05) (Figure 4(b)). These results suggest that mRNA expression levels of the ligands of DARC could also be reduced by Huaier aqueous extract, especially in MCF-7 cells.

### 3.4. Huaier Aqueous Extract Decreases the Secretion of DARC Ligands by MCF-7 and MDA-MB-231 Cells In Vitro

**Figure 5:**
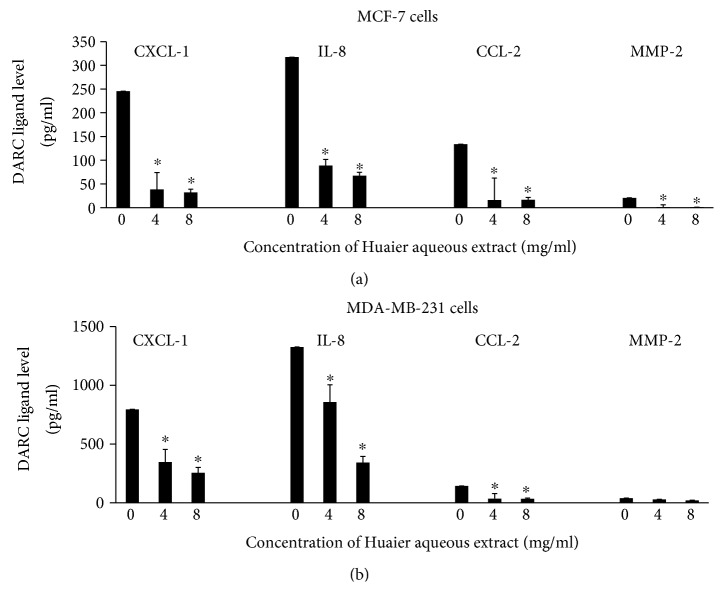
Effect of Huaier aqueous extract on the secretion of DARC ligands. The levels of CXCL-1, IL-8, CCL-2, and MMP-2 in conditioned medium of (a) MCF-7 cells and (b) MDA-MB-231 cells after the treatment of different concentrations (4 or 8 mg/ml) of Huaier aqueous extract for 48 h. The concentrations of secreted proteins were evaluated by ELISA. ^∗^*P* < 0.05 compared with values at 0 mg/ml.

To test the effect of Huaier aqueous extract (4 or 8 mg/ml) for 48 h on the secretion of DARC ligands in vitro, ELISA was carried out. The data showed that treatment with Huaier aqueous extract significantly reduced the secretion of DARC ligands compared with the control in both MCF-7 and MDA-MB-231 cells (*P* < 0.05) (Figures 5(a) and 5(b)). The results indicate that Huaier aqueous extract decreases the secretion of DARC ligands by MCF-7 and MDA-MB-231 cells in vitro.

## 4. Discussion

Duffy antigen is an antigenic determinant of the Duffy blood group system that is first found on vivax for its entry into human red blood cells [18]. Human DARC can selectively bind to proinflammatory CC, angiogenic ELR^+^ CXC (glutamic acid leucine-arginine+), and CXC chemokines with high affinity. Although DARC is structurally similar to other chemokine receptors, it does not induce G protein-coupled signal transduction or Ca^2+^. Therefore, DARC is unable to couple to signal transduction pathways activated by typical chemokine receptors when artificially expressed in in vitro cellular systems [19, 20]. DARC controls chemokine through different ways, including chemokine release into blood, posttranslational modifications of plasma chemokines, and DARC-independent removal mechanism [21, 22]. Since DARC binds with angiogenic CXC chemokines, as well as some CC chemokines, by preventing the biological effects of these chemokines, DARC can induce tumor necrosis and antimetastatic effect [23].

It is shown that DARC is involved as a negative regulator in breast cancers, mainly by sequestration of angiogenic chemokines and subsequent inhibition of tumor neovascularity. DARC overexpression induces inhibition of tumorigenesis and/or metastasis through interfering with tumor angiogenesis in vivo [10]. DARC is found to bind angiogenic ELR^+^ CXC chemokines CXCL-1 and IL-8 and play a negative role in tumor progression through the control of angiogenesis by reducing angiogenic ELR^+^ CXC chemokine secretion [24]. Another ligand, CCL-2, has been found to have the ability to accelerate tumor growth and metastasis of cancer cells upon binding with typical specific receptors. Overexpression of DARC in human breast cancer cells has been reported to downregulate CCL-2 levels and subsequently inhibit the proliferation and metastasis of breast cancer cells in vivo and in vitro [10]. MMP-2 plays a crucial role in the progression of breast cancer by degrading extracellular matrix (ECM) components [25]. MMP-2 is also an important ligand of DARC.

The absence of multiple atypical chemokine binders such as DARC, D6, and CCX-CKR, which is associated with higher VEGF and MMP-9 expression, predicts the presence and extent of ALN metastasis in breast cancer [26]. Moreover, DARC isoforms in the tumor microenvironment could reduce the levels of promalignant chemokines in different degrees, have differential effects on tumor growth and vascularization, and contribute to differential potential of metastasis [4]. In addition, the concentration of DARC in serum of patients is significantly correlated with the relapse risk of patients [27]. However, the different effects of DARC between primary and metastatic breast cancers have not been well known.

To investigate the expression of DARC in primary and metastatic breast cancer, 30 cases with recurrence and metastasis are enrolled. We have detected the expression of DARC in the patients using immunohistochemical staining. Our findings demonstrate that DARC is reduced at different stages of breast cancer.

There are many reasons for the different expressions of DARC in primary and metastatic breast cancer, including tumor heterogeneity, false positive or false negative reactions, and genetic drift or clonal selection [28]. In the present study, we also find changes of ER, PR, and HER2 expression in primary and metastatic tissues. However, there is no statistically significant difference (data was not shown). This result is in accordance with a meta-analysis reported in 2015 [29]. The reduced expression of DARC in metastatic or recurrent tissues may prompt an important role in breast cancer progression and have a relationship with tumor recurrence and metastasis. Since DARC expression is reduced, the related angiogenic chemokines may be overexpressed. Breast cancer cells may become active and metastasize to distant positions. Therefore, any drug that can promote DARC expression in breast cancer cells may reduce cancer metastasis.

In the treatment for cancers, TCM has become more and more popular for its convenient intaking, inexpensiveness, and less harm to normal cells. Huaier is a type of fungus that has been used in TCM for many years. The drug, which is already approved by the Chinese Food and Drug Administration, has been used in many kinds of cancer patients [30]. However, the effect of Huaier aqueous extract on DARC and its ligands has not been investigated.

Our experiment shows that Huaier aqueous extract not only improves the expression of DARC in vitro but also reduces the expression of its ligands such as CCL-2, IL-8, MMP-2, and CXCL-1, especially in MCF-7 cells. However, DARC is confirmed to be a powerful chemokine controller in posttranslational stages rather than in translational stages. Therefore, DARC does not affect chemokine mRNA expression commonly. Although the secretion of CCL-2, IL-8, MMP-2, and CXCL-1 is reduced in vitro, it is not definite that Huaier aqueous extract can reduce ligand secretion by promoting the expression of DARC. Since Huaier aqueous extract can cause apoptosis of cells, this may affect protein secretion.

In conclusion, our study demonstrates that expression of DARC is reduced in metastatic breast cancer tissues, and Huaier aqueous extract affects the expression of DARC and its ligands. However, the present study only included a limited number of patients, and more samples are needed for further investigations. Moreover, we will carry out in vivo experiments in the future.

## Figures and Tables

**Table 1:** General data of patients with primary tumor.

Age (years)	≤40	6
	≥40	24

Tumor size	T1	8
	T2	22

Lymph node metastasis	N0	12
	N1	6
	N2	7
	N3	5

Pathological grade	1	1
	2	15
	3	14

Clinical stage	IA	6
	IB	0
	IIA	9
	IIB	4
	IIIA	6
	IIIB	0
	IIIC	5

ER	Positive	19
	Negative	11

PR	Positive	16
	Negative	14

HER2	Positive	8
	Negative	22

**Table 2:** Specific primers of DARC and other relevant molecules.

H-CCL-2-F	ACAAGCAAACCCAAACTCCG
H-CCL-2-R	AAACAGGGTGTCTGGGGAAA
H-MMP-2-F	GGAAGTCTGTGTTGTCCAGAGG
H-MMP-2-R	CCAAGCGGTCTAAGTCCAGAG
H-CXCL1-F	CTGGCTTAGAACAAAGGGGCT
H-CXCL1-R	TAAAGGTAGCCCTTGTTTCCCC
h-IL-8(CXCL8)-f	GGTGCAGTTTTGCCAAGGAG
h-IL-8(CXCL8)-r	TGGGGTGGAAAGGTTTGGAG
H-ACTB-F-2	CATGTACGTTGCTATCCAGGC
H-ACTB-R-2	CTCCTTAATGTCACGCACGAT

**Table 3:** Expression score of DARC in primary and metastatic breast cancer tissues.

	Groups	*N*	Means	*P*
The score of DARC	Primary	30	6.7667	0.001
	Metastatic	30	3.1333	

## Data Availability

The data used to support the findings of this study are available from the corresponding author upon request.
